# Gene Expression Profile Change and Associated Physiological and Pathological Effects in Mouse Liver Induced by Fasting and Refeeding

**DOI:** 10.1371/journal.pone.0027553

**Published:** 2011-11-09

**Authors:** Fang Zhang, Xiang Xu, Ben Zhou, Zhishui He, Qiwei Zhai

**Affiliations:** Key Laboratory of Nutrition and Metabolism, Institute for Nutritional Sciences, Shanghai Institutes for Biological Sciences, Chinese Academy of Sciences, Shanghai, China; Rutgers University, United States of America

## Abstract

Food availability regulates basal metabolism and progression of many diseases, and liver plays an important role in these processes. The effects of food availability on digital gene expression profile, physiological and pathological functions in liver are yet to be further elucidated. In this study, we applied high-throughput sequencing technology to detect digital gene expression profile of mouse liver in fed, fasted and refed states. Totally 12162 genes were detected, and 2305 genes were significantly regulated by food availability. Biological process and pathway analysis showed that fasting mainly affected lipid and carboxylic acid metabolic processes in liver. Moreover, the genes regulated by fasting and refeeding in liver were mainly enriched in lipid metabolic process or fatty acid metabolism. Network analysis demonstrated that fasting mainly regulated Drug Metabolism, Small Molecule Biochemistry and Endocrine System Development and Function, and the networks including Lipid Metabolism, Small Molecule Biochemistry and Gene Expression were affected by refeeding. In addition, FunDo analysis showed that liver cancer and diabetes mellitus were most likely to be affected by food availability. This study provides the digital gene expression profile of mouse liver regulated by food availability, and demonstrates the main biological processes, pathways, gene networks and potential hepatic diseases regulated by fasting and refeeding. These results show that food availability mainly regulates hepatic lipid metabolism and is highly correlated with liver-related diseases including liver cancer and diabetes.

## Introduction

As the source of materials for basal metabolism, food availability plays an important role in the progression of diseases [1]. There are numerous reports on undernutrition in humans. For example, the classic Minnesota experiment assessed the physiological and psychological effects of severe food restriction and rehabilitation [2], and Dutch and Chinese data showed that prenatal exposure to famine increases risk of schizophrenia in later life [3], [4]. Throughout the 20th century, interest in food availability and human health changed from concerns on the physiology of undernutrition to increasing health problems associated with overnutrition [5]. Overnutrition-related chronic diseases, including cardiovascular diseases, obesity and diabetes, are becoming increasingly common in the world as a main result of changes in diet and physical activity [1], [6]. It was reported that an estimated 14% of US adults used fasting as a means to control body weight and this approach has long been advocated as an intermittent treatment for gross refractory obesity [7]. Meanwhile, calorie restriction and alternate-day fasting were reported to induce weight loss, extend lifespan and prevent the common chronic diseases in various species [8], [9], [10]. Food availability shows multiple physiological and pathological effects, therefore the molecular mechanisms associated with fasting and refeeding needs to be understood in depth.

Being the biggest endocrine gland in our body, liver has a central role in metabolism and plays a key role in the maintenance of nutrient homeostasis [11]. Many of the regulatory effects in response to diet initially occur in liver, which then modulates the activities of other organs in terms of nutrient utilization and metabolism [12]. Thus, the consequences of food availability in liver are wide spread. Fasting induces many changes especially lipid and glucose metabolism in liver, key among these changes are fatty acid oxidation and hepatic gluconeogenesis [13], [14]. The previous studies show that fasting induces peroxisome proliferator-activated receptor-α (PPARα), which stimulates the expression of the acyl-CoA dehydrogenases (ACDs) and other fatty acid oxidation genes [15], [16]. While, phosphoenolpyruvate carboxykinase (PEPCK), a key enzyme in hepatic gluconeogenesis, is induced by glucagon, catecholamines and glucocorticoids during periods of fasting, but is dominantly inhibited by glucose-induced increases in insulin secretion upon refeeding [17]. Some other hepatic gluconeogenesis controllers such as PGC1 and SIRT1, are altered by nutritional status and considered as drug targets for diabetes and obesity [18], [19]. The way to alter the key genes to keep metabolism homeostasis becomes popular in the treatment of chronic diseases. Therefore, the global gene profiling of liver under different nutritional status will be very helpful to elucidate the key genes and the gene networks regulated by food availability.

Microarray technology has been applied to study the genome-wide gene expression levels in mammalian tissues regulated by food availability [20]. Microarray expression analysis has been successfully used in the genomic profiling of short- and long-term caloric restriction effects in the liver of aging mice [21]. Similarly, starvation response in mouse liver showed strong correlation with life-span-prolonging processes induced by calorie restriction [22]. In chicken liver, microarray experiments revealed the central role of lipid and acetyl-CoA metabolisms and its regulation at transcriptional level in response to short-term fasting [23]. Recently, high-throughput sequencing is emerging as an attractive alternative to microarrays for transcriptome profiling, because high-throughput sequencing-based expression analysis shows major advances in robustness, resolution and reproducibility based on its relatively unbiased and direct digital readout [24], [25], [26]. In terms of both profiling coverage and quantitative accuracy of high throughput sequencing, the application of this new technology in transcriptome profiling altered by nutrient status is important to further understand the underlying mechanisms.

In this study, we measured the gene expression profile in feeding, fasting and refeeding mouse liver by high-throughput sequencing, and showed the main biological processes, pathways, networks and potential liver related diseases regulated by food availability.

## Results

### High throughput sequencing and the top abundant, abundance change and fold change genes in mouse liver regulated by food availability

**Figure 1:**
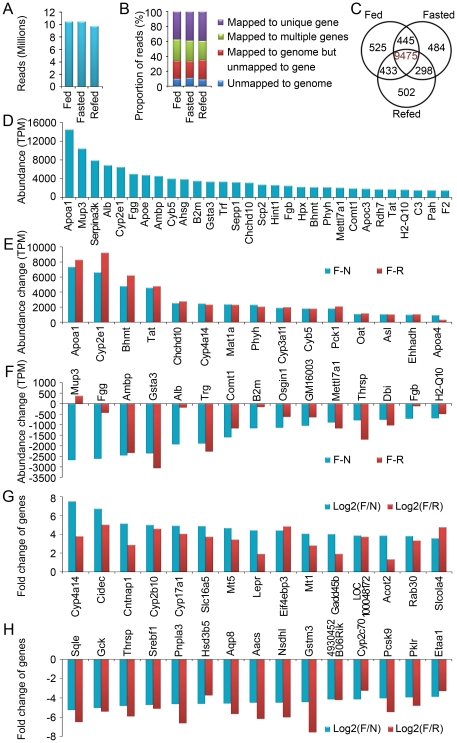
Sequencing and mapping messages of mouse liver mRNA profiling under feeding, fasting and refeeding conditions. (A) Reads of high quality clean tags from high-throughput sequencing experiments. Total liver RNA from C57BL/6 mice fed ad libitum with chow, fasted for 24 hr, or fasted for 24 hr and refed for 24 hr was used to prepare the high-throughput sequencing library. (B) Proportions of high quality clean tags unmapped and/or mapped to unique genes, multiple genes and genome. (C) Gene numbers between feeding, fasting and refeeding states. (D) The top 30 abundant genes in normal feeding mouse liver from the high-throughput sequencing were quantified and shown as transcripts per million (TPM). (E) The top 15 abundance change of genes upregulated by fasting, and their abundance change following refeeding. N, gene abundance under normal feeding condition; F, gene abundance under fasting condition; R, gene abundance under refeeding condition. (F) The top 15 abundance changes of genes downregulated by fasting, and their abundance change following refeeding. (G) The top 15 fold change of genes upregulated by fasting, and their fold change following refeeding. (H) The top 15 fold change of genes downregulated by fasting, and their fold change following refeeding.

To global survey the gene expressing pattern in mouse liver regulated by food availability, high throughput sequencing was applied in this study. Finally, we obtained about 10.48, 10.45 and 9.72 million reads of high quality clean tags from mice fed ad libitum, fasted and refed respectively (Figure 1A). In these high quality clean tags, averagely, 81.6%, 65.8% and 38.6% reads can be mapped to annotated mouse genome, genes, and unique genes respectively (Figure 1B). Totally 12162 genes were detected, and 10878, 10702 and 10708 unique genes were detected and quantified from fed, fasted and refed samples respectively, which shared 9475 genes in common (Figure 1C).

Based on the Solexa sequencing data, the top 30 highly expressed genes in normal feeding adult mouse liver were shown in Figure 1D. *Apoa1* gene encoding Apolipoprotein A-I, a major protein component of high density lipoprotein, was identified as the most abundant gene using this method. Furthermore, the top 15 abundance changes of genes upregulated by fasting were shown in Figure 1E. Interestingly, the abundance changes of these 15 genes upregulated by fasting were almost completely reversed by refeeding except *Apoa4* (Figure 1E). Even in the top 100 abundance changes of genes upregulated by fasting, 82 genes were almost completely reversed by refeeding. The top 15 abundance changes of genes downregulated by fasting were shown in Figure 1F. However the abundance changes of *Mup3*, *Fgg*, *Alb*, *B2m* and *Fgb* induced by fasting were not well reversed by refeeding (Figure 1F). In addition, we showed the top 15 fold changes of genes upregulated or downregulated by fasting in Figure 1G,H. 13 of the top 15 fold change genes upregulated by fasting were still upregulated after refeeding, however 12 of the top 15 fold change genes downregulated by fasting will be upregulated by refeeding compared to normal feeding. The above results show that the upregulated or downregulated genes in top abundance or fold change have different interesting dynamic pattern and might have critical functions in adaption to food availability.

To understand the final effects of refeeding on gene expression, the top 15 abundance or fold change genes upregulated or downregulated by refeeding compared to normal feeding were shown in Figure S2A,B.

### Differentially expressed genes and clustering

Among the 10878, 10702 and 10708 unique genes detected from fed, fasted and refed samples, differentially expressed genes between these three states were quantified and shown in Figure S3A. To avoid the possible noise signal from high-throughput sequencing, the genes with average TPM (transcripts per million) less than 1 in these three states were excluded. The remained 8815 genes and their abundance were shown in Table S2, and were used to calculate the fold changes and false discovery rate (FDR). In this study, the absolute fold change no less than 1.5 and FDR less than 0.001 were used to define the differentially expressed genes. According to this definition, totally 2305 genes were differentially expressed between fed, fasted and refed states (Figure S3A). There were 1509 and 1180 genes significantly affected by fasting and refeeding respectively compared to normal feeding, and 384 genes regulated by fasting were not well reversed by refeeding (Figure S3B).

To gain insights into the 2305 differentially expressed genes regulated by food availability, we divided them into 8 clusters based on the different dynamic pattern induced by fasting and refeeding (Figure S3C). Cluster 1 to 3 contained the genes upregulated by fasting, which is named as Cluster A. Cluster 4 to 6 contained genes downregulated by fasting, which is named as Cluster B. Cluster 7 and 8 included the genes unaffected by fasting but regulated by refeeding.

### The main biological processes and pathways regulated by fasting and refeeding

**Figure 2:**
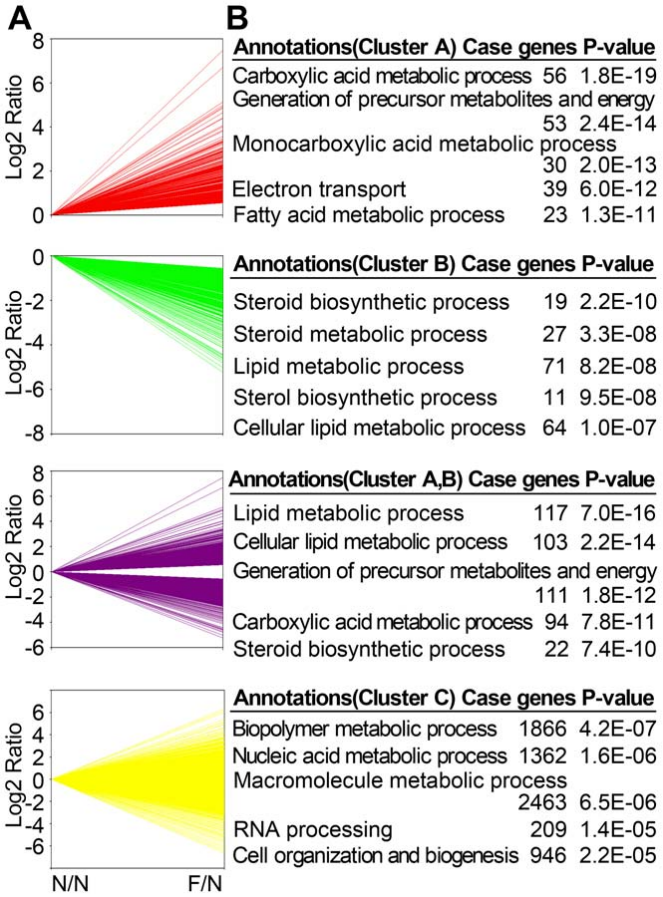
Genes and the related biological processes in mouse liver regulated by fasting. (A) The 8815 selected genes as described in Materials and Methods and Figure S3A were separated into three distinct clusters according to the genes upregulated, downregulated, or unaffected by fasting compared with normal feeding. Red lines indicate Cluster A including 472 genes upregulated by fasting. Green lines indicate Cluster B including 1037 genes downregulated by fasting. Purple lines indicate 1509 genes affected by fasting, which include all genes in Cluster A and B. Yellow lines indicate Cluster C including 7306 genes unaffected by fasting. N, gene abundance under normal feeding condition; F, gene abundance under fasting condition. (B) The clustered genes were assigned to different biological processes based on Gene Ontology using the web tool DAVID. The top 5 biological functions and the case genes in each cluster ranked by *P*-value were listed (*P*<0.001, case genes ≥10).

To identify the possible biologic functions regulated by fasting, the 8815 genes were divided into Cluster A, B and C with 472 genes upregulated, 1037 genes downregulated and 7306 genes unaffected by fasting respectively (Figure 2A). Based on Gene Ontology analysis, Cluster A genes mainly enriched in carboxylic acid metabolic process, especially in its sub-process monocarboxylic acid or even more specifically in fatty acid metabolic process (Figure 2B). Cluster A genes also enriched in generation of precursor metabolites and energy, especially in its sub-process electron transport. Cluster B genes mainly enriched in lipid metabolic process, especially in its sub-process steroid metabolic process typically including steroid and sterol biosynthetic processes (Figure 2B). Fasting-affected 1509 genes including Cluster A and Cluster B mainly enriched in lipid and carboxylic acid metabolic processes, generation of precursor of metabolites and energy (Figure 2B). Cellular lipid metabolic process and steroid biosynthetic process in lipid metabolic process were also enriched. These results show that fasting mainly affect lipid and carboxylic acid metabolic processes in liver, but will not affect some hepatic basic biological processes including biopolymer, nucleic acid and macromolecule metabolic processes.

Furthermore, Ingenuity and KEGG pathway analysis were performed to further elucidate the biological functions of the gene clusters regulated by fasting. The top 5 Ingenuity and KEGG pathways significantly affected by fasting were shown in Table 1 with associated genes. These pathway analysis data further confirmed that lipid and carboxylic acid metabolic processes in liver, especially fatty acid metabolism, were the main biological processes regulated by fasting.

**Table 1:** The top 5 Ingenuity and KEGG pathways and the associated genes significantly affected by fasting.

	Ingenuity Pathway		KEGG Pathway	
**Cluster A**472 genes	**Fatty Acid Metabolism**		**Fatty acid metabolism**	
	ACAD11-ACADL-ACADM-ACADVL-ACAT1-ACOX1-ACSL1-ALDH3A2-CPT2-CPT1A-CYP2E1-DCI-ECH1-EHHADH-HSD17B4-HSD17B10-PECI-SDS-SLC27A1-SLC27A2P = 6.24E-10		CPT2-TEKT3-ACSL1-ACADL-RGL3-ACADM-ACADVL-ACOX1-CUEDC2-ALDH3A2-CPT1A-KCNK4-CACNG8-DCI-HSD17B10-HSD17B4-PECI-EHHADH-OLFR916-ACAT1-CYP4A14P = 1.45E-11	
	**Valine, Leucine and Isoleucine Degradation**		**PPAR signaling pathway**	
	ACAD11-ACADL-ACADM-ACADVL-ACAT1-ALDH3A2-ECH1-EHHADH-HMGCL-HSD17B4-HSD17B10-SDSP = 6.78E-07		CPT2-TEKT3-ACSL1-PCK1-IFNE-SLC27A2-ANGPTL4-ACADL-RGL3-ACADM-ACOX1-CUEDC2-APOA1-FABP7-CPT1A-KCNK4-CACNG8-CYP7A1-ZSCAN10-CYP8B1-PPARA-ZFP831-SLC27A1-APOA5-EHHADH-OLFR916-CYP4A14P = 5.69E-10	
	**LPS/IL-1 Mediated Inhibition of RXR Function**		**Linoleic acid metabolism**	
	ABCC2-ABCC3-ACOX1-ACSL1-ALDH3A2-APOC4-CPT2-CPT1A-CYP7A1-FMO1-GSTT2-JUN-LBP-NR1I2-NR1I3-PAPSS2-PPARA-SLC27A1-SLC27A2-SULT1A1P = 1.23E-06		AGXT-AA960436-ABAT-GOT1-PCX-LINGO4-GPT-ASL-ASNS-VWA3B-GPT2P = 6.15E-06	
	**Propanoate Metabolism**		**Alanine and aspartate metabolism**	
	ACAD11-ACADL-ACADM-ACADVL-ACAT1-ACSL1-ALDH3A2-ECH1-EHHADH-SDS-SUCLG1P = 1.64E-06		CYP2C37-CYP2E1-FCRLA-CYP2C39-CYP3A11-GPR124-CYP3A13-CYP3A16-ZC3H10-CCR1L1-CYP3A25-PLA2G12A-CYP2C50P = 1.84E-05	
	**β-alanine Metabolism**		**Citrate cycle (TCA cycle)**	
	ACAD11-ACADL-ACADM-ACADVL-ALDH3A2-DPYD-ECH1-EHHADH-SDSP = 5.60E-06		PCK1-IFNE-ACO2-MIS12-FH1-A830018L16RIK-PCX-LINGO4-SLC32A1-SUCLG1-FAM154A-DLSTP = 1.24E-04	
**Cluster B** **1037 genes**	**Biosynthesis of Steroids**		**Complement and coagulation cascades**	
	CYP26A1-DHCR7-HMGCR-IDI1-LSS-SQLEP = 6.16E-04		C9-HDDC3-FGA-TIFA-FGG-C1QA-FAM114A1-C1QC-C4BP-1110007C09RIK-C6-1110012L19RIK-CD59A-FAM125A-CFH-TXLNA-F2R-F3-CFB-HC-KNG1-MBL2-OLFR129-CPB2-F11-C8B-C8AP = 4.75E-07	
	**Endometrial Cancer Signaling**		**Nucleotide sugars metabolism**	
	CASP9-CCND1-CDH1-CTNNB1-GRB2-MAPK6-PIK3R1-PTEN-RRAS2P = 7.37E-04		HSD3B7-AKR1E1-HSD17B12-AKR1C6-GM6897-RDH11-OLFR382-UGDH-GALE-OLFR1246-UGP2P = 1.01E-06	
	**Colorectal Cancer Metastasis Signaling**		**Biosynthesis of steroids**	
	BAX-CASP9-CCND1-CDH1-CTNNB1-FZD7-GRB2-LRP1-MAPK6-MMP15-PIK3R1-RELA-RHOC-RHOU-RND3-RRAS2-TGFB1-TNFRSF1A-VEGFBP = 2.60E-03		SC5D-DHCR7-HMGCR-LSS-GPSM1-SQLE-MVD-IDI1P = 1.33E-05	
	**ILK Signaling**		**Glycine, serine and threonine metabolism**	
	CCND1-CTNNB1-DSP-FERMT2-MAPK6-MYL9-PARVA-PIK3R1-PTEN-RELA-RHOC-RHOU-RND3-TNFRSF1A-VEGFBP = 5.50E-03		ALAS2-CBS-SHROOM1-6720468P15RIK-CHKA-HSD3B7-GAMT-PEMT-AKR1E1-HSD17B12-AKR1C6-GM6897-TARS-RDH11-OLFR382P = 3.46E-04	
	**IL-8 Signaling**		**Metabolism of xenobiotics by cytochrome P450**	
	BAX-CCND1-CDH1-GNA13-GNAI3-KDR-MAPK6-NOX4-PIK3R1-RELA-RHOC-RHOU-RND3-RRAS2-VEGFBP = 7.42E-03		CYP2C70-ADH1-ATAD4-CYP1A2-FAM71F1-GSTA3-GSTA4-RASGEF1B-GSTM6-ADH4-GSTM7-UGT2B1-UGT2A1-CYP2C44-GSTM3P = 3.81E-03	
**Fasting affected 1509 genes**				
	**Fatty Acid Metabolism**	P = 3.48E-12	**Fatty acid metabolism**	P = 3.32E-08
	**PXR/RXR Activation**	P = 3.17E-08	**Linoleic acid metabolism**	P = 3.13E-07
	**LPS/IL-1 Mediated Inhibition of RXR Function**	P = 5.44E-07	**Glycine, serine and threonine metabolism**	P = 1.04E-06
	**Tryptophan Metabolism**	P = 6.22E-07	**Metabolism of xenobiotics by cytochrome P450**	P = 3.54E-06
	**Metabolism of Xenobiotics by Cytochrome P450**	P = 2.17E-06	**PPAR signaling pathway**	P = 1.01E-05

**Figure 3:**
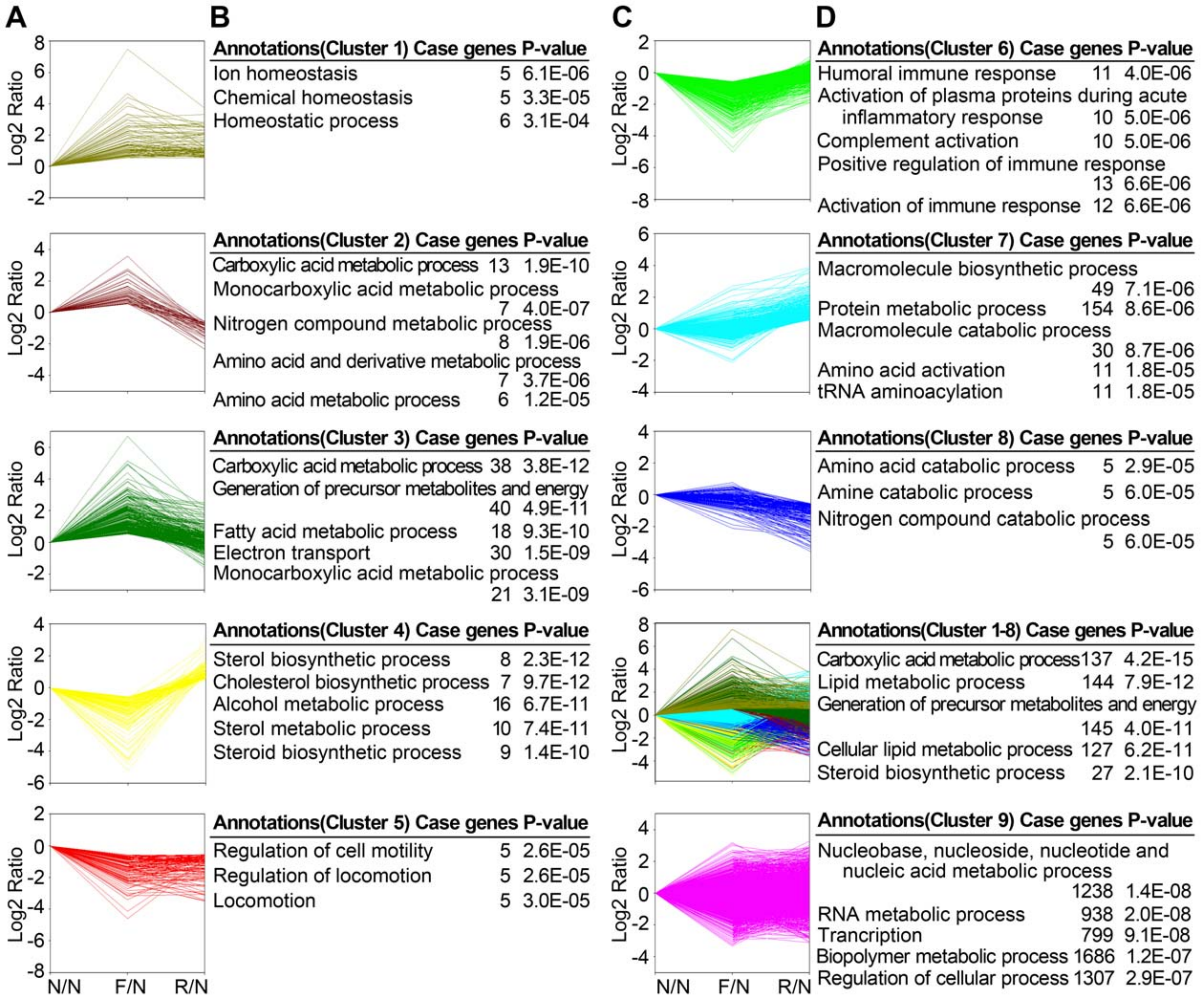
Genes and the related biological processes in mouse liver regulated by fasting and refeeding. (A, C) The 8815 selected genes as described in Materials and Methods and Figure S3A were separated into nine distinct clusters according to the genes upregulated, downregulated or unaffected by fasting and refeeding compared with normal feeding. Cluster 1 included 83 genes upregulated by fasting and refeeding. Cluster 2 included 50 genes upregulated by fasting and downregulated by refeeding. Cluster 3 included 339 genes upregulated by fasting and unaffected by refeeding. Cluster 4 included 128 genes downregulated by fasting and upregulated by refeeding. Cluster 5 included 123 genes downregulated by fasting and refeeding. Cluster 6 included 786 genes downregulated by fasting and unaffected by refeeding. Cluster 7 included 618 genes unaffected by fasting and upregulated by refeeding. Cluster 8 included 178 genes unaffected by fasting and downregulated by refeeding. Cluster 9 included 6510 genes unaffected by fasting and refeeding. N, gene abundance under normal feeding condition; F, gene abundance under fasting condition; R, gene abundance under refeeding condition. (B, D) The clustered genes were assigned to different biological processes based on Gene Ontology using the web tool DAVID. The top biological processes and the case genes in each cluster ranked by *P*-value were listed (*P*<0.001, case genes ≥5).

To identify the possible biologic functions regulated by both the whole fasting and refeeding process, the 8815 genes were divided into nine clusters according to the dynamic patterns upregulated, downregulated or unaffected by fasting or refeeding as shown in Figure 3A,C. Among the fasting-affected 1509 genes, fasting-upregulated 472 genes were divided into Cluster 1, 2, 3, and fasting-downregulated 1037 genes were divided into Cluster 4, 5, 6, according to their expression upregulated, downregulated or unaffected by refeeding. 384 genes in Cluster 1, 2, 4 and 5 were significantly regulated by fasting, and none of them recovered to normal feeding levels after refeeding (Figure 3A). Interestingly, Cluster 1 genes are enriched in ion and chemical homeostasis and homeostatic process (Figure 3B); Cluster 2 genes are mainly enriched in carboxylic acid metabolic process, especially in its sub-process monocarboxylic acid metabolic process including amino acid metabolic process; Cluster 4 genes are enriched in alcohol and sterol metabolic processes and steroid biosynthetic process, especially in sterol or more specifically in cholesterol biosynthetic process; Cluster 5 genes are enriched in regulation of cell motility and locomotion. 1125 genes in Cluster 3 and Cluster 6 were significantly upregulated or downregulated by fasting, but almost completely recovered to normal feeding levels after refeeding (Figure 3A,C). Cluster 3 mainly enriched in carboxylic acid metabolic process and generation of precursor metabolites and energy, and Cluster 6 genes mainly enriched in immune responses (Figure 3B,D). These data suggest that immune responses, carboxylic acid metabolic process and generation of precursor metabolites and energy are significantly regulated by fasting, but can be recovered to normal feeding states after refeeding. Fasting-unaffected 7306 genes were divided into Cluster 7, 8, 9 according to their expression upregulated, downregulated or unaffected by refeeding. 796 genes in Cluster 7 and 8 were not directly affected by fasting, but were upregulated or downregulated respectively after refeeding (Figure 3C). Cluster 7 genes are enriched in macromolecule biosynthetic process, protein metabolic process and macromolecule catabolic process (Figure 3D), and Cluster 8 genes are enriched in amino acid, amine and nitrogen compound catabolic processes. These data demonstrate the genes regulated by fasting or refeeding with similar dynamic pattern are usually enriched in similar feature biological processes affected by food availability. Furthermore, all the differentially expressed genes are mainly enriched in carboxylic acid and lipid metabolic processes and generation of precursor metabolites and energy (Figure 3).

Moreover, Ingenuity and KEGG pathway analysis showed that the different gene clusters with different dynamic patterns regulated by fasting and refeeding usually enriched in different pathways, and all the differentially expressed genes are mainly enriched in the pathways of fatty acid metabolism and metabolism of xenobiotics by cytochrome P450 (Table S1).

### The main networks regulated by fasting and refeeding

**Figure 4:**
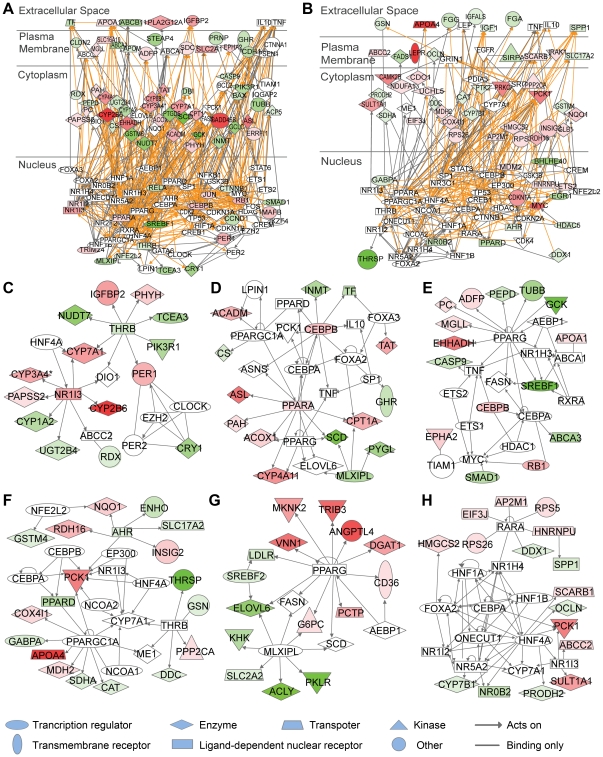
Network representation of the biological processes in mouse liver regulated by fasting and refeeding. (A) The top 5 connected networks in 1125 genes upregulated or downregulated by fasting and recovered to normal feeding states after refeeding. The gene networks were analyzed by Ingenuity. Genes upregulated or downregulated by fasting are represented in red or green color respectively. The top 3 networks were shown in (C), (D) and (E). (C) Network of Drug Metabolism, Small Molecule Biochemistry and Endocrine System Development and Function. (D) Network of Lipid Metabolism, Small Molecule Biochemistry and Molecular Transport. (E) Network of Lipid Metabolism, Molecular Transport and Small Molecule Biochemistry. (B) The top 5 connected networks of 1180 genes upregulated or downregulated after refeeding compared to normal feeding. The top 3 networks were shown in (F), (G) and (H). (F) Network of Lipid Metabolism, Small Molecule Biochemistry and Gene Expression. (G) Network of Lipid Metabolism, Small Molecule Biochemistry and Molecular Transport. (H) Network of Hepatic System Disease, Lipid Metabolism and Molecular Transport.

To further understand the global gene changes during fasting-refeeding process, we sought to computationally decipher the principle networks regulated by fasting and refeeding using Ingenuity. The top 5 gene networks regulated by fasting were combined and shown in Figure 4A, including Drug Metabolism, Small Molecule Biochemistry, Endocrine System Development and Function (Figure 4C), Lipid Metabolism, Small Molecule Biochemistry, Molecular Transport (Figure 4D), and Lipid Metabolism, Molecular Transport, Small Molecule Biochemistry (Figure 4E). The top 5 gene networks significantly regulated by refeeding compared to normal feeding were shown in Figure 4B, including Lipid Metabolism, Small Molecule Biochemistry, Gene Expression (Figure 4F), Lipid Metabolism, Small Molecule Biochemistry and Molecular Transport (Figure 4G), Hepatic System Disease, Lipid Metabolism and Molecular Transport (Figure 4H). The network analysis demonstrates that Lipid Metabolism, Small Molecule Biochemistry and Molecular Transport are the three main sub-networks regulated by fasting and refeeding. In addition, the sub-network Hepatic System Disease regulated by refeeding implicates that fasting and refeeding can significantly affect the development of hepatic diseases.

### The potential hepatic diseases affected by fasting and refeeding

**Figure 5:**
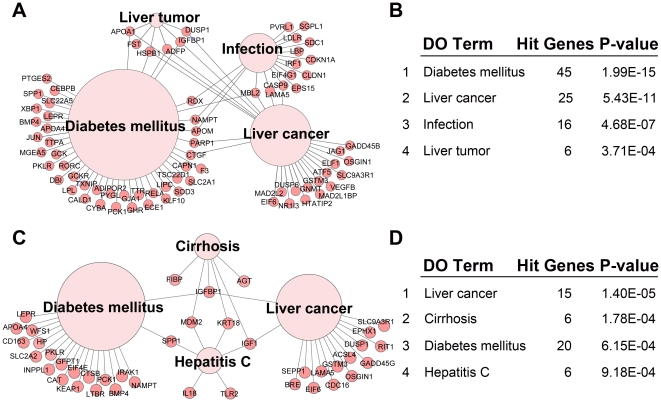
Genes regulated by fasting or refeeding linked to different liver diseases. (A) The map of top 4 liver diseases enriched with the genes regulated by fasting. 1509 genes upregulated or downregulated by fasting were assigned to different diseases using the web tool FunDO. The sizes of the disease nodes are proportional to the number of enriched genes. (B) The number of hit genes and *P*-value of the top 4 enriched liver diseases in (A). (C) The map of top 4 liver diseases enriched with the genes upregulated or downregulated after refeeding. 1180 genes affected by refeeding were assigned to different diseases using the web tool FunDO. (D) The number of hit genes and *P*-value of the top 4 enriched liver diseases in (C).

To further elucidate the correlation between food availability and hepatic diseases, we assigned the hepatic genes regulated by fasting and refeeding to different diseases using web tool FunDO. As shown in Figure 5A,B, 1509 genes regulated by fasting were mainly related with diabetes mellitus, liver cancer, infection and liver tumor. Fasting-affected genes enriched in the indicated diseases were listed in Table S3. In addition, the 1180 genes significantly affected by refeeding compared to normal feeding were enriched in liver cancer, cirrhosis, diabetes mellitus and hepatitis C (Figure 5C,D). Refeeding-affected genes enriched in the indicated diseases were listed in Table S4. These results demonstrate that food availability is significantly correlated with the development of liver cancer and diabetes mellitus.

## Discussion

This study provides the basic gene expression profile data of feeding, fasting and refeeding mouse liver by high throughput sequencing, and demonstrates the main biological processes, pathways, networks and potential liver related diseases affected by fasting and refeeding. Food availability mainly regulates lipid metabolism, especially fatty acid metabolism in liver, and is significantly correlated with some liver related diseases including liver cancer and diabetes. These results should be very helpful to further understand the metabolism and diseases in liver regulated by food availability.

High throughput RNA sequencing greatly increases our ability to quantitatively detect mRNA level with relatively unbiased measurements of gene abundance [25], [27]. With this technology, we showed the gene expression data of mouse liver under feeding, fasting and refeeding conditions in Table S2. The top 30 abundant genes were shown in Figure 1. According to the mouse array data collected in BioGPS (http://biogps.gnf.org) [28], 24 genes of the top 30 abundant genes are specifically or most highly expressed in liver. The rest 6 genes, including *B2m*, *Chchd10*, *Hint1*, *Phyh*, *Mettl7a1* and *Pah* are also highly expressed in liver when analyzed by BioGPS. These results show that our data are comparable with the previous array data. A common feature of liver is the key metabolic center. By Gene Ontology analysis [29], [30], we found 21 genes among the top 30 abundant genes are enriched in metabolic process, which is consistent with the feature of liver as the key metabolic center. In the top 30 abundant genes, most of them are well known for their important roles in liver. However the role of a few genes including *Chchd10* and *Mettl7a1* in liver is yet to be elucidated. In the top 30 abundant genes, 18 of them are in the top 30 abundance change genes affected by fasting as shown in Figure 1. 17 genes among the top 30 abundance change genes affected by fasting can be enriched in metabolic process. Similarly, among the top 30 fold change genes affected by fasting, 19 genes can be clustered in metabolic process. For example, *Gck* and *Pklr* among the 19 genes encoding hexokinase and pyruvate kinase respectively, are the key genes involved in glycolysis process [14], [31]. It has been reported that the flux through glycolysis process is attenuated in response to fasting [32]. Consistently, we found that fasting significantly downregulated *Gck* and *Pklr* (Fig. 1H). In addition, *Cyp4a14 and slc16a5*, as the top 30 fold change genes induced by 24-h fasting (Figure 1G), have been reported to be involved in hypertension and the disposition of various drugs respectively [33], [34], [35]. However, the role of *Cyp4a14 and slc16a5* in fasting response is still largely unknown, and whether *Cyp4a14 and slc16a5* are involved in the major pathways including glucose and lipid metabolism regulated by fasting needs to be studied in the future. In conclusion, these data show that the top abundance change and fold change genes induced by fasting are mainly involved in liver metabolic process. Further studies focused on these top abundance change or fold change genes, especially some largely unknown genes, will be very helpful to understand the gene network and the related physiological and pathological processes in liver.

It has been shown that hepatic fatty acid, amino acid and glucose metabolism were altered by fasting [22], [23], [36], [37]. Similarly, we found that fasting significantly affected fatty acid and amino acid metabolism in liver using Gene Ontology analysis and KEGG and Ingenuity pathway analysis with the digital gene expression data from high-throughput sequencing (Figure 2 and Table 1). In terms of fatty acid metabolism, previous studies show that fasting induces PPARα, which stimulates the expression of ACDs and other fatty acid oxidation genes [15], [16]. Moreover, fasting upregulates fatty acid oxidation flux [32], [38]. Similarly, our quantitative data showed that fasting upregulated mRNA levels of *PPARα* and *Acadm* to 1.8- and 3.0-fold respectively. Besides, we showed fasting-upregulated genes enriched in fatty acid metabolism in Table 1, including *Cpt1a*, encoding the rate-limiting enzyme in fatty acid oxidation, and *Ehhadh*, encoding the key enzyme catalyzing two steps in fatty acid oxidation [39], [40]. In terms of amino acid metabolism, previous studies showed that fasting significantly affected genes involved in amino acid metabolism [37], and increased amino acid feeding into TCA cycle [32]. Similarly, our data showed that fasting-upregulated genes are enriched in valine, leucine and isoleucine degradation, alanine and aspartate metabolism and other amino acids metabolism (Table 1). It is reported that liver starts to generate glucose from carbon-3 compounds derived from fatty acid and amino acid as substrates during fasting [14]. Metabolic fluxes studies showed that glucose production from glycolysis was decreased and limited and absolute gluconeogenesis were markedly increased in response to fasting [41]. Liver played an important role in keeping glucose homeostasis mainly through downregulating glycolytic reactions, upregulating gluconeogenic reactions and reducing glycogenolysis flux significantly [32], [41]. Consistently, our data showed that fasting dramatically downregulated the mRNA level of pyruvate kinase (*Pklr*), a key enzyme in glycolysis [14], to 6.6% (Table S2). Meanwhile fasting upregulated the mRNA level of PEPCK (*Pck1*), a key regulatory enzyme in gluconeogenesis [42], to 3.8-fold. And the mRNA level of glycogen phosphorylase (*Pygl*), which catalyzes the rate-limiting step in glycogenolysis, was downregulated by fasting to 30% (Table S2). Taken together, our data provide the detailed quantitative gene expression change involved in the metabolic changes in response to fasting, such as the reverse of glycolysis to gluconeogenesis, upregulation of fatty acid and amino acid metabolism for gluconeogenesis (Figure 2,3, and Table 1). The observed gene expression changes are in line with previous studies on metabolic fluxes, and provide new insights into how gene expression is regulated to meet the metabolic changes in response to fasting. It is reported that refeeding syndrome is caused by rapid refeeding after a period of starvation. Refeeding syndrome featured with hypophosphataemia is caused by rapid refeeding after a period of starvation, but the underlying molecular mechanism is unclear [43], [44]. Here, we studied the refeeding effect at gene expression level, and we found that 1180 genes were significantly changed after refeeding (see Cluster 1, 2, 4, 5, 7 and 8 in Figure 3. and Table S2). Our results showed that refeeding mainly affected amino acid, fatty acid and steroid metabolism in liver (Figure 3. and Table S1), which might provide the underlying molecular mechanism of refeeding syndrome. As shown in Figure 3, food availability triggers a dynamic change in metabolic pathways and is a good model for understanding how these pathways are mutually organized.

Adult humans often undertake acute fasts for cosmetic, religious or medical reasons. For example, it has been reported that fasting was used as a means to control body weight and has long been advocated as an intermittent treatment for obesity [5]. Hence, the studies on fasting associated diseases are in great need for human health. Previous study reported that starvation response in mouse liver shows strong correlation with life-span-prolonging processes [22]. Using the web tool FunDO, we found diabetes mellitus, liver cancer and infection are highly correlated with fasting (Figure 5), suggesting a molecular connection between fasting and hepatic diseases. As shown in Table S3, among the 45 genes related to diabetes mellitus, 17 and 28 genes were upregulated and downregulated by fasting respectively. Among the 28 downregulated genes, glycogen phosphorylase (*Pygl*) catalyzes the rate-limiting step in the degradation of glycogen in animals [14], and glycogen phosphorylase inhibitors has been proposed as potential antidiabetic agents [45]. Similarly, thyroid hormone-binding protein transthyretin (*Ttr*) downregulated by fasting (Table S3), is increased in insulin-resistant mice, and lowing thyroid hormone-binding protein levels may enhance insulin sensitivity in type 2 diabetes [46]. These examples show that the expression changes of some genes regulated by fasting are beneficial for diabetes. However, glucokinase (*Gck*) downregulated by fasting (Table S3), has been identified as a promising drug target for type 2 diabetes through its activators [47]. Therefore, fasting induced the expression change of some genes might leading to the development of type 2 diabetes. To further evaluate the effect of fasting on diabetes mellitus and other diseases, mRNA and protein levels, and even enzyme activity are required to be combined for detailed evaluation. Furthermore, other fasting ways, such as long-term fasting and calorie restriction, need to be used for future studies. In addition, liver cancer, cirrhosis and diabetes mellitus is highly correlated with refeeding (Figure 5). Taken together, these results provide a broad view of genes, food availability and their correlation with diseases, and whether fasting is good for diseases treatment needs further studies.

In summary, we have generated a quantitative gene profiling in feeding, fasting and refeeding mouse liver with high throughput sequencing. Our results demonstrated food availability induced dynamic changes of feature biological processes, pathways and networks. Food availability mainly regulated lipid metabolism, especially fatty acid metabolism in liver, and is highly correlated with some liver-related diseases including liver cancer and diabetes. These quantitative results should be very helpful to further understand the metabolism and diseases in liver regulated by food availability.

## Materials and Methods

### Animal experiments

All animal experimental procedures were approved by the Institutional Animal Care and Use Committee of the Institute for Nutritional Sciences (Protocol number 2007-AN-9). C57BL/6 male mice at the age of 7 weeks purchased from SLAC (Shanghai, China) were randomly divided into three groups for 12 mice per group, and were allowed to have access to water and diets ad libitum. At the age of 9 weeks, mice fed ad libitum, fasted for 24 h, or fasted for 24 h and then refed for 24 h were sacrificed about 3 h after the beginning of light cycle, and the livers were immediately removed and snap-frozen in liquid nitrogen.

### Sample preparation and solexa library construction

Liver samples were ground in liquid nitrogen, and total RNA was isolated using Trizol reagent (Invitrogen). The high quality of total RNA was confirmed by Bioanalyzer 2100 (Agilent). Solexa libraries were constructed following the manufacturer's standard according to the schematic shown in Figure S1.

### Solexa sequencing and data analysis

The image files generated by from Illumina 1G sequencer were processed to produce digital-quality sequence data. Then the high quality reads were screened from the original data, and the adaptors were removed from each sequence. Finally, high quality clean tags were compared with RefSeq database (released at Feb 9, 2009) and the expression level of each gene was normalized to transcripts per million (TPM). The significance of digital gene expression profiles were analyzed as described previously [48]. To avoid the potential noise signal from high-throughput sequencing, we excluded the genes with average TPM less than 1 in these three states. The remained 8815 genes were used to calculate the fold changes and false discovery rate (FDR), which is adjusted *p*-values based on ordered *p*-values for several thousands of genes testing [49]. In this study, based on the assumption that the majority of genes are not changed [22], the absolute fold change no less than 1.5 and FDR less than 0.001 were used to define the differentially expressed genes including the upregulated and downregulated genes. The genes upregulated, downregulated and unaffected by fasting were classified into Cluster A, B and C respectively. Cluster A, B and C were further divided into 9 groups including Cluster 1–3, 4–6 and 7–9 respectively, according to the upregulated, downregulated or unaffected effect of refeeding. The expression pattern of these genes were visualized using the heat-map function in the R base package [50].

### Biological process, pathway and network analysis

Gene Ontology constitutes a controlled vocabulary of about 20,000 terms organized in three independent hierarchies for cellular components, molecular functions, and biological processes (www.geneontology.org) [51]. The clustered genes were assigned to biological processes based on Gene Ontology using the web tool DAVID (http://david.abcc.ncifcrf.gov/home.jsp) [29], [30]. Hypergeometric test was used as the statistical method to select enriched biological process Gene Ontology terms for each cluster. The pathways involved in these gene clusters were analyzed by KEGG (www.genome.jp/kegg/pathway.html) [52] and Ingenuity (Ingenuity Systems Inc.) Network analysis was also performed with Ingenuity.

### FunDo analysis

To study gene-disease relationships, the differentially expressed genes regulated by fasting or refeeding were assigned to different diseases based on Disease Ontology and peer-reviewed evidence from GeneRIF using the web tool FunDO (http://django.nubic.northwestern.edu/fundo/) [53]. Then Cytoscape v2.6.2 was used to visualize gene-disease interaction networks [54].

## Supporting Information

Figure S1 Schematic of the protocol for constructing the high-throughput sequencing library from liver total RNA. Magnetic oligo(dT) beads were used to isolate poly(A) mRNA from the total RNA samples. cDNA was synthesized from the isolated mRNA using random hexamer primers. Then the cDNA was digested with Dpn II, and the standard Solexa protocol for digital gene expression-tag profiling was followed thereafter to create cDNA libraries.
(TIF)

Figure S2 Top reads and fold change genes upregulated or downregulated by refeeding compared to normal feeding. (A) The top 15 abundance change of genes upregulated or downregulated by refeeding. (B) The top 15 fold change of genes upregulated or downregulated by refeeding.
(TIF)

Figure S3 Genes differentially expressed in mouse liver between feeding, fasting and refeeding states. (A) 8815 genes with average TPM no less than 1 in the feeding, fasting and refeeding samples were selected to analyze the gene expression profile. Number of genes differentially expressed between fasting and feeding states or between refeeding and feeding states according to the indicated fold change and FDR value was listed. (B) Venn diagram for the 2305 differentially expressed genes with absolute fold change ≥1.5 and FDR<0.001. (C) Heat-map images for the 2305 differentially expressed genes. The selected genes were classified into Cluster 1 to 8, based on the genes upregulated, downregulated, or unaffected by fasting and/or refeeding. Cluster A and Cluster B included upregulated and downregulated genes respectively induced by fasting. Red and blue indicate genes with high and low abundance respectively.
(TIF)

Table S1 The top 5 Ingenuity and KEGG pathways and the associated genes significantly affected by fasting and refeeding.
(DOC)

Table S2 Gene expression data of mouse liver under feeding, fasting and refeeding conditions. The 8815 genes with average TPM no less than 1 in these three states were listed. N_TPM, the TPM of the indicated genes under normal feeding condition; F_TPM, the TPM of the indicated genes under fasting condition; R_TPM, the TPM of the indicated genes under Refeeding condition.
(XLS)

Table S3 List of fasting-affected genes enriched in the correlated liver diseases.
(XLS)

Table S4 List of Refeeding-affected genes enriched in the correlated liver diseases.
(XLS)
