# Supplementary figures and images for: Gene Expression Profile Change and Associated Physiological and Pathological Effects in Mouse Liver Induced by Fasting and Refeeding
Source: PLoS One. 2011 Nov 9;6(11):e27553. doi: 10.1371/journal.pone.0027553 (PMC3212576; doi:10.1371/journal.pone.0027553)

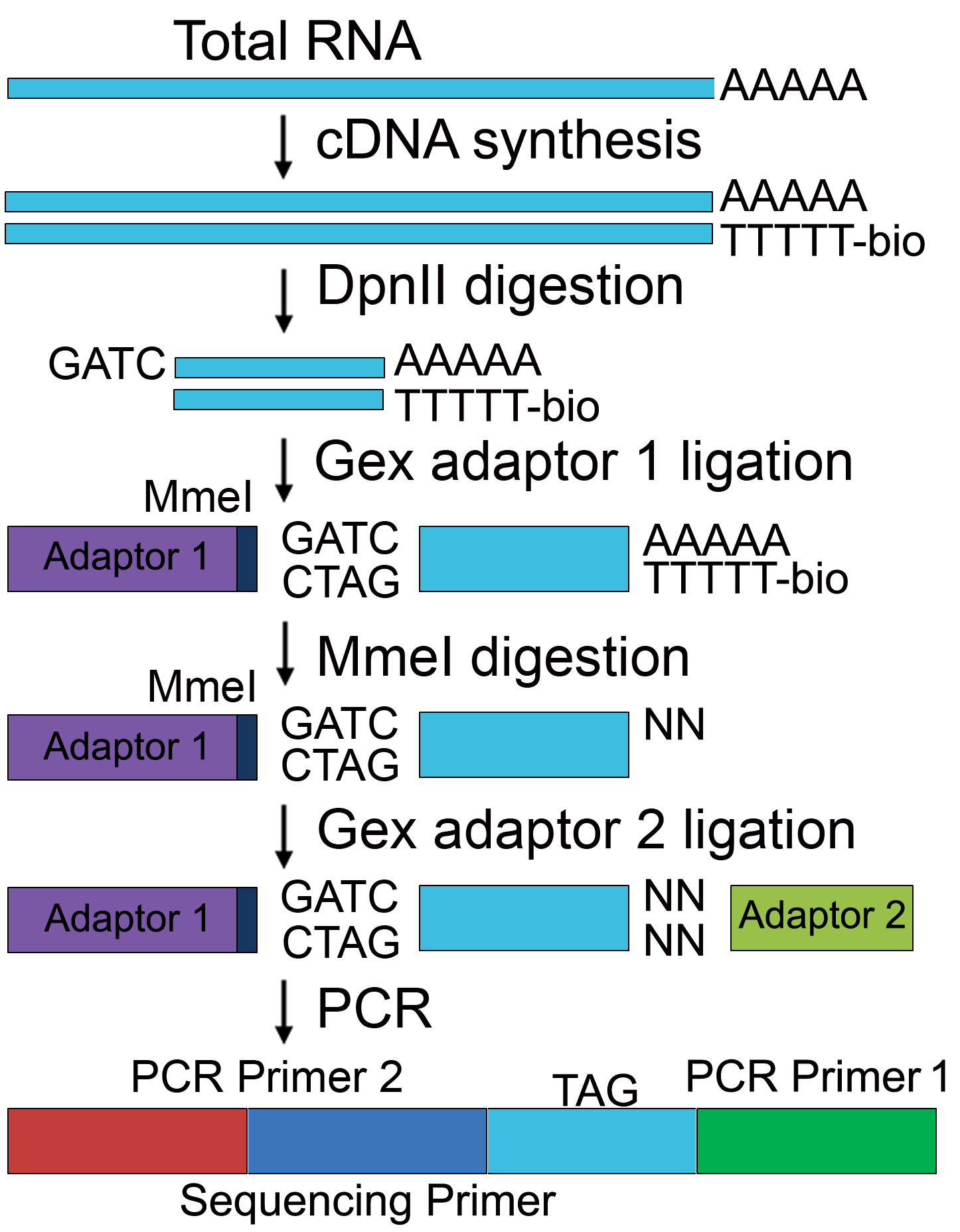

Supplement: Figure S1 — Schematic of the protocol for constructing the high-throughput sequencing library from liver total RNA. Magnetic oligo(dT) beads were used to isolate poly(A) mRNA from the total RNA samples. cDNA was synthesized from the isolated mRNA using random hexamer primers. Then the cDNA was digested with Dpn II, and the standard Solexa protocol for digital gene expression-tag profiling was followed thereafter to create cDNA libraries. (TIF) [file pone.0027553.s001.tif]

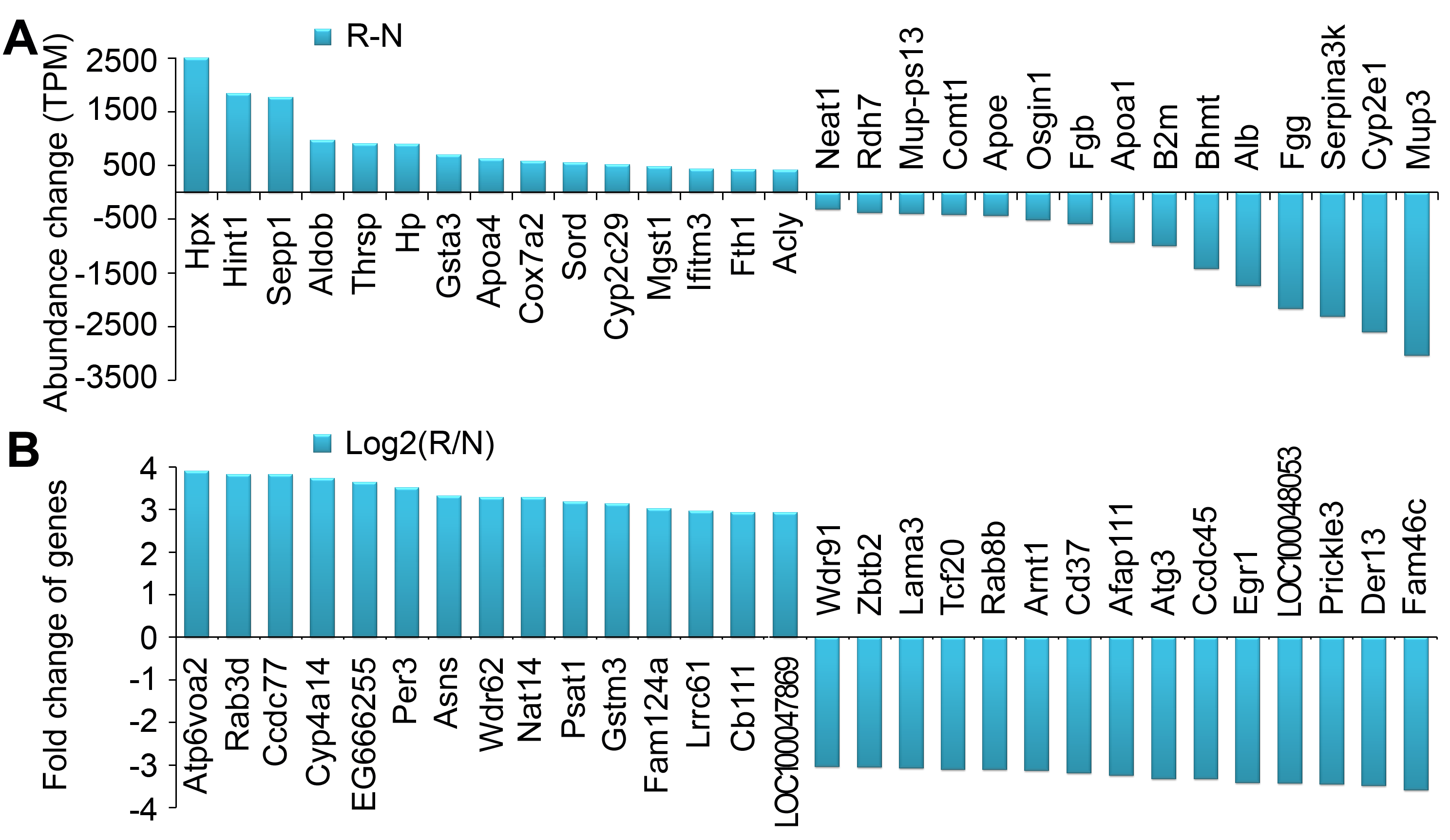

Supplement: Figure S2 — Top reads and fold change genes upregulated or downregulated by refeeding compared to normal feeding. (A) The top 15 abundance change of genes upregulated or downregulated by refeeding. (B) The top 15 fold change of genes upregulated or downregulated by refeeding. (TIF) [file pone.0027553.s002.tif]

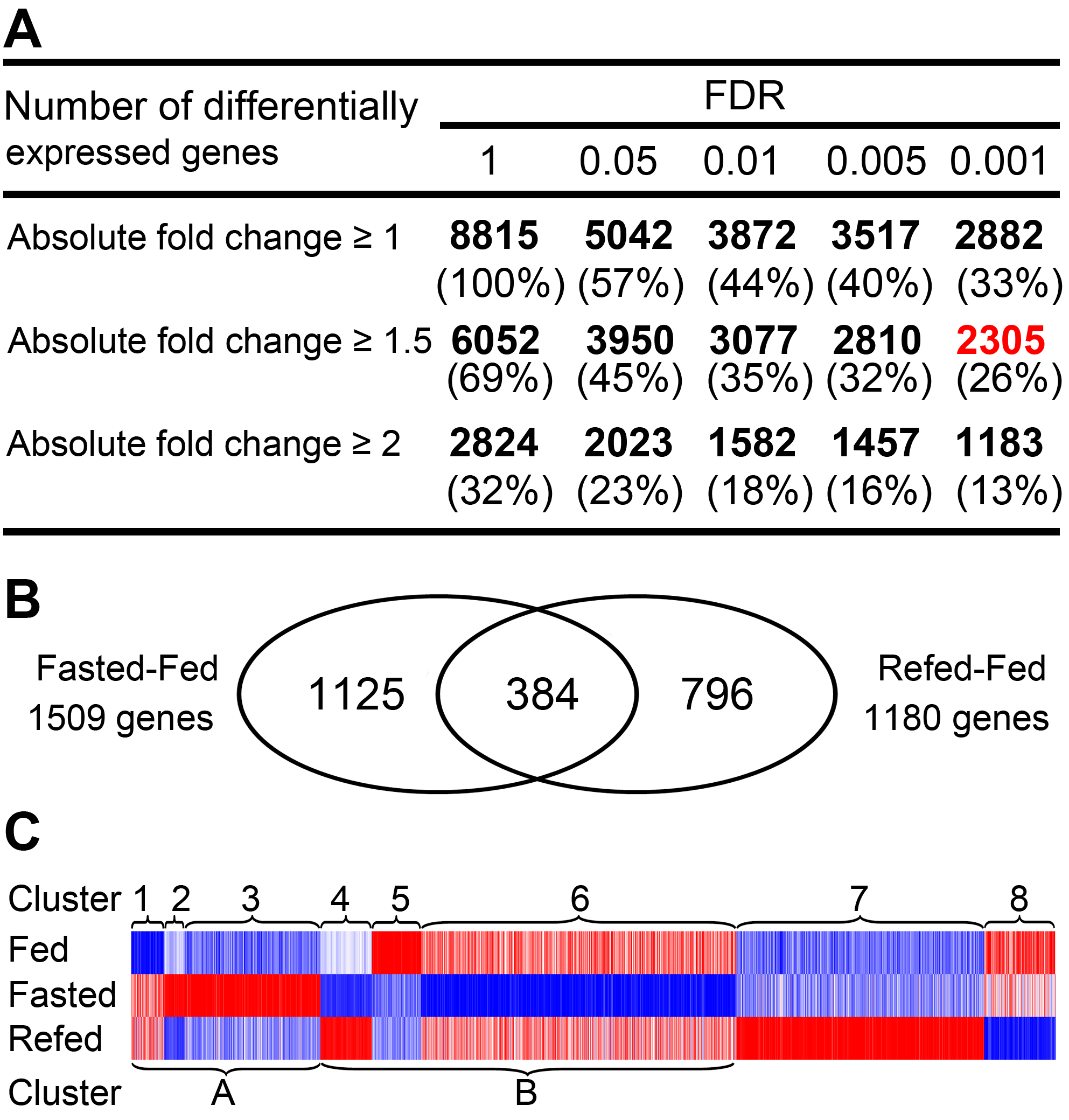

Supplement: Figure S3 — Genes differentially expressed in mouse liver between feeding, fasting and refeeding states. (A) 8815 genes with average TPM no less than 1 in the feeding, fasting and refeeding samples were selected to analyze the gene expression profile. Number of genes differentially expressed between fasting and feeding states or between refeeding and feeding states according to the indicated fold change and FDR value was listed. (B) Venn diagram for the 2305 differentially expressed genes with absolute fold change ≥1.5 and FDR<0.001. (C) Heat-map images for the 2305 differentially expressed genes. The selected genes were classified into Cluster 1 to 8, based on the genes upregulated, downregulated, or unaffected by fasting and/or refeeding. Cluster A and Cluster B included upregulated and downregulated genes respectively induced by fasting. Red and blue indicate genes with high and low abundance respectively. (TIF) [file pone.0027553.s003.tif]
