# Supplementary material for: Gene Expression Profile Change and Associated Physiological and Pathological Effects in Mouse Liver Induced by Fasting and Refeeding
Source: PLoS One. 2011 Nov 9;6(11):e27553. doi: 10.1371/journal.pone.0027553 (PMC3212576; doi:10.1371/journal.pone.0027553)
Supplement: Table S1 — The top 5 Ingenuity and KEGG pathways and the associated genes significantly affected by fasting and refeeding. (DOC) [file pone.0027553.s004.doc]

***Table S1.*** *The top 5 Ingenuity and KEGG pathways and the associated genes significantly affected by fasting and refeeding.*

|  | **Ingenuity Pathway** | **KEGG Pathway** |
| --- | --- | --- |
| **Cluster 1**  **83 genes** | **NRF2-mediated Oxidative Stress Response** | **Alanine and aspartate metabolism** |
|  | DNAJB4-DNAJB9-GSTT2-HERPUD1-PRKCA-PTPLAD1  P = 1.48E-04 | ASNS-GPT2  P = 1.80E-03 |
|  | **Xenobiotic Metabolism Signaling** |  |
|  | ABCC2-CAMK2B-GSTT2-MED1-PRKCA-SCAND1  P = 8.20E-04 |  |
|  | **RAR Activation** |  |
|  | MED1-PRKCA-RDH16-SCAND1  P = 6.76E-03 |  |
|  | **Endothelin-1 Signaling** |  |
|  | EDNRB-MYC-PRKCA-TMEM87B  P = 7.38E-03 |  |
|  | **Neuregulin Signaling** |  |
|  | MYC-NRG4-PRKCA  P = 8.78E-03 |  |
| **Cluster 2**  **50 genes** | **Fatty Acid Metabolism** | **PPAR signaling pathway** |
|  | ACSL1-CPT2-CYP2C19-CYP2E1-CYP3A43-CYP4F8-SLC27A2  P = 7.49E-08 | CPT2-ACSL1-PCK1-SLC27A2-ANGPTL4  P = 1.92E-03 |
|  | **LPS/IL-1 Mediated Inhibition of RXR Function** |  |
|  | ACSL1-CPT2-CYP2C19-LBP-SLC27A2-SLCO1A2-SULT1A1  P = 1.58E-06 |  |
|  | **Metabolism of Xenobiotics by Cytochrome P450** |  |
|  | CYP2C19-CYP2E1-CYP3A43-CYP4F8-GSTT3  P = 1.6E-05 |  |
|  | **Arachidonic Acid Metabolism** |  |
|  | CYP2C19-CYP2E1-CYP3A43-CYP4F8-EPHX2  P = 3.59E-05 |  |
|  | **Tryptophan Metabolism** |  |
|  | AFMID-CYP2C19-CYP2E1-CYP3A43-CYP4F8  P = 4.21E-05 |  |
| **Cluster 3**  **339 genes** | **Fatty Acid Metabolism** | **Fatty acid metabolism** |
|  | ACAD11-ACADL-ACADM-ACADVL-ACAT1-ACOX1-ALDH3A2-CPT1A-CYP2B6-CYP2C19-CYP2C39-CYP3A4-CYP3A7-CYP3A43-CYP4A11-DCI-ECH1-EHHADH-HSD17B4-HSD17B10-PECI-SDS-SLC27A1  P = 2.99E-17 | ACADL-ACADM-ACADVL-ACOX1-ALDH3A2-CPT1A-DCI-HSD17B10-HSD17B4-PECI-EHHADH-ACAT1  P = 8.33E-10 |
|  | **LPS/IL-1 Mediated Inhibition of RXR Function** | **PPAR signaling pathway** |
|  | ABCC3-ACOX1-ALDH3A2-APOC4-CPT1A-CYP2B6-CYP2C19-CYP3A4-CYP3A7-CYP4A11-CYP7A1-FABP7-FMO1-FMO2-JUN-NR1I2-NR1I3-PAPSS2-PPARA-SLC27A1-SULT1D1  P = 4.34E-11 | ACADL-ACADM-ACOX1-APOA1-FABP7-CPT1A-CYP7A1-CYP8B1-PPARA-SLC27A1-APOA5-EHHADH  P = 1.63E-06 |
|  | **PXR/RXR Activation** | **gamma-Hexachlorocyclohexane degradation** |
|  | ABCC3-ALDH3A2-CPT1A-CYP2B6-CYP2C19-CYP3A4-CYP3A7-CYP7A1-NR1I2-NR1I3-PAPSS2-PPARA P = 1.39E-09 | ALPL-CYP3A11-CYP3A13-CYP3A16-CYP3A25  P = 8.52E-05 |
|  | **Valine, Leucine and Isoleucine Degradation** | **Linoleic acid metabolism** |
|  | ACAD11-ACADL-ACADM-ACADVL-ACAT1-ALDH3A2-ECH1-EHHADH-HMGCL-HSD17B4-HSD17B10-SDS  P = 5.27E-09 | CYP2C39-CYP3A11-CYP3A13-CYP3A16-CYP3A25-PLA2G12ACYP2C50  P = 1.0E-04 |
|  | **Tryptophan Metabolism** | **Valine, leucine and isoleucine degradation** |
|  | ACAT1-ALDH3A2-AS3MT-CYP2B6-CYP2C19-CYP2C39-CYP3A4-CYP3A7-CYP3A43-ECH1-EHHADH-HSD17B4-HSD17B10-SDS-TDO2  P = 9.23E-08 | ACADM-ALDH3A2-HSD17B10-HMGCL-HSD17B4-EHHADH-ACAT1  P = 1.75E-04 |
| **Cluster 4**  **128 genes** | **Biosynthesis of Steroids** | **Biosynthesis of steroids** |
|  | DHCR7-HMGCR-IDI1-LSS-MVD-SQLE  P = 6.07E-08 | DHCR7-HMGCR-LSS-SQLE-MVD-IDI1  P = 1.34E-07 |
|  | **Nucleotide Sugars Metabolism** | **Nucleotide sugars metabolism** |
|  | GALE-UGDH-UGP2  P = 1.19E-04 | RDH11-UGDH-GALE-UGP2  P = 6.76E-05 |
|  | **Butanoate Metabolism** | **Reductive carboxylate cycle (CO2 fixation)** |
|  | AACS-ACAT2-ALDH1A7-BDH2-ELOVL6  P = 1.22E-04 | IDH1-ACSS2-ACLY  P = 2.07E-04 |
|  | **Inositol Metabolism** |  |
|  | DHCR24-FADS1-LSR-RDH11-SC4MOL  P = 1.55E-04 |  |
|  | **Glutathione Metabolism** |  |
|  | ACSS2-GSTM3-IDH1-PGD  P = 7.41E-04 |  |
| **Cluster 5**  **123 genes** | **C21-Steroid Hormone Metabolism** | **Glycine, serine and threonine metabolism** |
|  | CYP21A2-HSD3B5-HSD3B7  P = 2.82E-04 | ALAS2-CBS-CHKA-HSD3B7  P = 7.31E-04 |
|  | **Acute Phase Response Signaling** | **C21-Steroid hormone metabolism** |
|  | C9-FGA-FGG-HAMP-SAA4  P = 5.17E-03 | CYP21A1-HSD3B5  P = 8.90E-04 |
|  | **Androgen and Estrogen Metabolism** |  |
|  | HSD3B5-HSD3B7-UGT2B17  P = 8.13E-03 |  |
|  | **Glycine, Serine and Threonine Metabolism** |  |
|  | ALAS2-CBS-CHKA  P = 8.9E-03 |  |
|  | **Methionine Metabolism** |  |
|  | CBS-CCBL1  P = 1.37E-02 |  |
| **Cluster 6**  **786 genes** | **Complement System** | **Complement and coagulation cascades** |
|  | C5-C6-C1QA-C1QC-C8A-C8B-CFB-CFH-MBL2  P = 6.64E-06 | C1QA-C1QC-C4BP-C6-CD59A-CFH-F2R-F3-CFB-HC-KNG1-MBL2-CPB2-F11-C8B-C8A  P = 3.12E-07 |
|  | **Apoptosis Signaling** | **Alkaloid biosynthesis II** |
|  | BAX-BCL3-CAPN1-CAPN2-CAPN7-CASP9-GAS2-MAPK6-PARP1-RAF1-RELA-RRAS2-TNFRSF1A-XIAP  P = 1.87E-05 | CES1-ES22-EG13909-AADAC-ES31  P = 1.22E-03 |
|  | **Endometrial Cancer Signaling** | **Metabolism of xenobiotics by cytochrome P450** |
|  | CASP9-CCND1-CDH1-CTNNB1-GRB2-MAPK6-PIK3R1-PTEN-RAF1-RRAS2  P = 7.57E-05 | ADH1-CYP1A2-GSTA3-GSTA4-GSTM6-ADH4-GSTM7-UGT2B1-UGT2A1-CYP2C44  P = 2.04E-03 |
|  | **Antigen Presentation Pathway** | **Colorectal cancer** |
|  | B2M-HLA-A-HLA-C-HLA-DQA1-MR1-TAP2-TAPBP  P = 9.84E-05 | BAX-CASP9-CTNNB1-CCND1-FZD1-FZD7-GRB2-MSH2-PIK3R1-TGFB1-RAF1  P = 2.96E-03 |
|  | **Metabolism of Xenobiotics by Cytochrome P450** |  |
|  | ADH4-ADH1C-AKR1C4-CYP1A2-CYP2C44-CYP2J9-CYP4F12-GSTA3-GSTA4-GSTM2-GSTM6-UGT2A1-UGT2B4-UGT2B7  P = 1.06E-04 |  |
| **Cluster 7**  **618 genes** | **Polyamine Regulation in Colon Cancer** | **Proteasome** |
|  | PSMA4-PSMB1-PSMB2-PSMB3-PSMB4-PSMB7-PSMC2-PSMC3-PSMC4-PSMC5-PSMC6-PSMD1-PSMD3-PSMD6-PSMD8-PSMD12-PSMD13-PSMD14  P = 1.07E-13 | PSMB1-PSMB4-PSMB7-PSMC2-PSMC3-PSMC5-PSMD3-PSMC4-PSMD13-PSMA4-PSMB2-PSMB3-PSMD8-PSMD14-PSMD6-PSMD12-PSMC6  P = 2.17E-17 |
|  | **Protein Ubiquitination Pathway** | **Aminoacyl-tRNA biosynthesis** |
|  | MDM2-PSMA4-PSMB1-PSMB2-PSMB3-PSMB4-PSMB7-PSMC2-PSMC3-PSMC4-PSMC5-PSMC6-PSMD1-PSMD3-PSMD6-PSMD8-PSMD12-PSMD13-PSMD14-TCEB2-UBE2D2-UBE2M-UCHL5-USO1-USP36  P = 2.39E-10 | SARS-VARS-FARSB-NARS-SARS2-IARS-GARS  P = 2.54E-05 |
|  | **Aminoacyl-tRNA Biosynthesis** | **Glycine, serine and threonine metabolism** |
|  | FARSA-FARSB-GARS-IARS-NARS-RARS2-SARS-SARS2-VARS-YARS2  P = 6.89E-04 | DLD-SARS-SARS2-PSPH-PSAT1-SHMT2-CHDH-AGXT2-GARS  P = 4.94E-05 |
|  | **Fructose and Mannose Metabolism** | **Carbon fixation** |
|  | ALDOA-ALDOB-DUSP16-NUDT5-SORD-TPI1  P = 2.62E-03 | ALDOA-MDH2-RPIA-TPI1-ALDOB  P = 4.70E-04 |
|  | **NRF2-mediated Oxidative Stress Response** | **Glycan structures - degradation** |
|  | BACH1-CCT7-CUL3-EPHX1-FTH1-GSTM4-KEAP1-MAFF-MAFK-MGST1-MGST3-NQO1-TXN  P = 4.21E-03 | AGA-ARSB-GLB1-MAN2B2-GALNS  P = 1.21E-03 |
|  |  | **Citrate cycle (TCA cycle)** |
|  |  | DLD-IDH3G-MDH2-SDHA-IDH2  P = 1.22E-03 |
| **Cluster 8**  **178 genes** | **Bile Acid Biosynthesis** | **Histidine metabolism** |
|  | CYP27A1-CYP7B1-SLC27A5  P = 6.24E-03 | DDC-FTCD-UROC1  P = 1.64E-03 |
|  | **FXR/RXR Activation** |  |
|  | CYP27A1-G6PC-SCARB1-SLC27A5  P = 7.44E-03 |  |
|  | **Histidine Metabolism** |  |
|  | CYP7B1-DDC-FTCD-UROC1  P = 7.64E-03 |  |
| **Differentially expressed 2305 genes** | | |
|  | **Fatty Acid Metabolism**  P = 3.02E-13 | **Glycine, serine and threonine metabolism**  P = 4.19E-12 |
|  | **LPS/IL-1 Mediated Inhibition of RXR Function**  P = 1.35E-10 | **Metabolism of xenobiotics by cytochrome P450**  P = 2.31E-08 |
|  | **Metabolism of Xenobiotics by Cytochrome P450**  P = 6.20E-10 | **Proteasome**  P = 6.42E-08 |
|  | **PXR/RXR Activation**  P = 5.81E-09 | **Citrate cycle (TCA cycle)**  P = 4.53E-07 |
|  | **Polyamine Regulation in Colon Cancer**  P = 1.42E-07 | **Fatty acid metabolism**  P = 9.81E-07 |
